# Genome Sequence of the First Coleopteran Iflavirus Isolated from Western Corn Rootworm, *Diabrotica virgifera virgifera* LeConte

**DOI:** 10.1128/genomeA.01530-16

**Published:** 2017-02-09

**Authors:** Sijun Liu, Yuting Chen, Thomas W. Sappington, Bryony C. Bonning

**Affiliations:** a Department of Entomology, Iowa State University, Ames, Iowa, USA; b Corn Insects & Crop Genetics Research Unit, USDA, ARS, Ames, Iowa, USA

## Abstract

The genome sequence of a novel iflavirus was identified from the transcriptome of the western corn rootworm, *Diabrotica virgifera virgifera*. The RNA sequence consists of 9,823 nucleotides (nt) with a 3′ polyadenylated tail, containing a single open reading frame that encodes a 3,028-amino-acid polyprotein.

## GENOME ANNOUNCEMENT

Western corn rootworm (WCR), *Diabrotica virgifera virgifera* LeConte (Coleoptera: Chrysomelidae), is the worst insect pest of maize in North America and is invasive in Europe (1). Management of WCR in the United States depends primarily on transgenic plants that express *Bacillus thuringiensis* (Bt) insecticidal toxins, but populations resistant to Bt toxins have been reported (2). Hence, novel control strategies for WCR management are needed. Small RNA viruses of insects, such as iflaviruses, have potential for insect pest management. Several iflaviruses have recently been discovered in the insect orders Hemiptera (3), Lepidoptera (4), and Hymenoptera (5–7). Here, we report the genome sequence of an iflavirus from WCR, the first identified from Coleoptera.

WCR adults were collected from cornfields in the United States (Iowa, Arizona, and Pennsylvania), and Europe (Hungary, Croatia, and Austria). Total RNA was extracted from ~100 individuals from different locations using TRIzol reagent (Life Technologies, Inc.). Additionally, putative viral RNA was isolated from virions, which were crudely extracted from WCR using the PEG virus precipitation kit (BioVision). Eight RNA sequencing (RNA-seq) libraries of either total RNA or viral RNA were prepared using the TruSeq RNA sample preparation kit version 2 (Illumina). All libraries were sequenced using an Illumina HiSeq 2000 platform, generating 3.7 to 21 million single-end 100-bp reads. After trimming adaptor sequences, the Illumina reads were *de novo* assembled using Trinity (8). Contigs (≥200 nucleotides [nt]) were used for BLAST annotation against the NCBI nonredundant (nr) protein database. We identified a 9,823-nt contig showing 27% amino acid sequence identity to its closest homolog, the polyprotein of an iflavirus, *Sacbrood virus* (accession number NP_049374).

This new virus, tentatively named *Diabrotica virgifera virgifera virus 1* (DvvV1), contains an open reading frame (ORF; nt 318 to 9401), with a 317-nt 5′-untranslated region (UTR) and a 422-nt 3′-UTR, followed by a polyadenylated tail. The G+C content of the virus is 39.45%. The entire ORF encodes a 3,028-amino-acid polyprotein. Conserved domains include four structural protein domains at the N terminus (nt 236 to 399, 314 to 454, 598 to 767, and 596 to 772) and two nonstructural protein domains at the C terminus, the RNA_helicase domain (nt 1423 to 1530) and RNA-dependent RNA polymerase domain (nt 2632 to 2965). Although the primary cysteine protease sequence motif of GxCG was identified (amino acids 2382 to 2385), the protease-C3 domain was not, suggesting divergence of the DvvV1 protease domain sequence. The presence of the virus in WCR adults was confirmed by reverse transcription-PCR (RT-PCR) using SuperScript III (Life Technologies, Inc.), with overlapping primers, and rapid amplification of cDNA ends (SMARTer RACE cDNA amplification kit; Clontech). Based on the arrangement of conserved domains, we conclude that this virus is a novel insect iflavirus. Relatively few iflaviruses are associated with overt symptoms or acute disease (6, 7), with deformed wing virus of honey bees being an exception (9). Although adult WCR with deformed wings have been observed in the field, it is not known whether this phenotype results from iflavirus infection.

### Accession number(s).

The genome sequence was deposited in GenBank under accession no. KY064174.
